# Expanding behavior pattern sensitivity analysis with model selection and survival analysis

**DOI:** 10.1186/s12917-018-1674-y

**Published:** 2018-11-20

**Authors:** Casey L. Cazer, Victoriya V. Volkova, Yrjö T. Gröhn

**Affiliations:** 1 Department of Population Medicine and Diagnostic Sciences, College of Veterinary Medicine, Cornell University, Ithaca, NY USA; 2 Department of Diagnostic Medicine/Pathobiology, College of Veterinary Medicine, Kansas State University, Manhattan, KS USA

**Keywords:** Sensitivity analysis, Antimicrobial resistance, Antibiotic resistance, Beef cattle, Behavior pattern, Linear regression, Survival analysis

## Abstract

**Background:**

Sensitivity analysis is an essential step in mathematical modeling because it identifies parameters with a strong influence on model output, due to natural variation or uncertainty in the parameter values. Recently behavior pattern sensitivity analysis has been suggested as a method for sensitivity analyses on models with more than one mode of output behavior. The model output is classified by behavior mode and several behavior pattern measures, defined by the researcher, are calculated for each behavior mode. Significant associations between model inputs and outputs are identified by building linear regression models with the model parameters as independent variables and the behavior pattern measures as the dependent variables. We applied the behavior pattern sensitivity analysis to a mathematical model of tetracycline-resistant enteric bacteria in beef cattle administered chlortetracycline orally. The model included 29 parameters related to bacterial population dynamics, chlortetracycline pharmacokinetics and pharmacodynamics. The prevalence of enteric resistance during and after chlortetracycline administration was the model output. Cox proportional hazard models were used when linear regression assumptions were not met.

**Results:**

We have expanded the behavior pattern sensitivity analysis procedure by incorporating model selection techniques to produce parsimonious linear regression models that efficiently prioritize input parameters. We also demonstrate how to address common violations of linear regression model assumptions. Finally, we explore the semi-parametric Cox proportional hazards model as an alternative to linear regression for situations with censored data. In the example mathematical model, the resistant bacteria exhibited three behaviors during the simulation period: (1) increasing, (2) decreasing, and (3) increasing during antimicrobial therapy and decreasing after therapy ceases. The behavior pattern sensitivity analysis identified bacterial population parameters as high importance in determining the trajectory of the resistant bacteria population.

**Conclusions:**

Interventions aimed at the enteric bacterial population ecology, such as diet changes, may be effective at reducing the prevalence of tetracycline-resistant enteric bacteria in beef cattle. Behavior pattern sensitivity analysis is a useful and flexible tool for conducting a sensitivity analysis on models with varied output behavior, enabling prioritization of input parameters via regression model selection techniques. Cox proportional hazard models are an alternative to linear regression when behavior pattern measures are censored or linear regression assumptions cannot be met.

## Background

Mathematical models are commonly used to understand and study biological systems that are complex to replicate and describe in individual laboratory or field studies. Models facilitate testing hypotheses that may be difficult or unethical to test in vivo, identification of critical parameters within a system, and they guide hypothesis generation and experimental design. Different types of mathematical models have improved our understanding of important issues in veterinary medicine, including infectious disease management [1–4], drug pharmacokinetics [5–8], and antimicrobial resistance [9–12]. With the rise in computing power, models have evolved to be more complex, detailed, and data-intensive. Simple susceptible-infectious-recovered models [12, 13] have given way to meta-population models [1, 4, 9] and now agent-based models [2, 14, 15], in which individuals (animals, bacteria, etc.) are modeled with unique characteristics and behaviors [16]. However, each layer of complexity adds additional sources of uncertainty to the model outputs, particularly when parameter values are from varied sources or unavailable and when the modeler must make assumptions about model structure and parameter values.

A robust sensitivity analysis defends a mathematical model against the adverse effects of excessive uncertainty. In short, sensitivity analysis attempts to identify how input parameter natural variation and/or uncertainty affects model output [17, 18]. A modeler can use sensitivity analysis to achieve many goals, including elimination of uninfluential parameters (simplification), model structure and code validation, improved understanding of the modelled system, and prioritization of the most influential parameters [17, 18]. The first sensitivity analysis techniques developed compared changes in one input parameter at a time to changes in the model output and hence were termed ‘local’ or ‘one-at-a-time’ approaches. The local parameter influence can be evaluated directly via partial derivatives [18] or statistically with a correlation coefficient [19]. In contrast, ‘global’ sensitivity analysis considers the entire domain for all input parameters and assess how changes in each input affect the model output after accounting for the effects of the other inputs [18]. Correlation coefficients and regression models (regressing model output on inputs) are commonly used for global sensitivity analysis, including decomposition of output variance and Taguchi designs [17, 18, 20]. Regression approaches are employed in sensitivity analyses because the regression coefficients can be used to rank model input parameters in their effect on the outputs [18]; such regression models may be viewed as meta-models used for investigating statistical associations between the output and parameter values in mathematical models [17, 18]. These techniques rely upon the modeler defining a numerical model output of interest and choosing a single time-point value of the output variable as the dependent variable (e.g., maximum or minimum value). Importantly, standard sensitivity analysis methods do not account for different output behaviors produced by one model and in some instances the behavior is of greater interest than a single time-point output value [20].

**Fig. 1:**
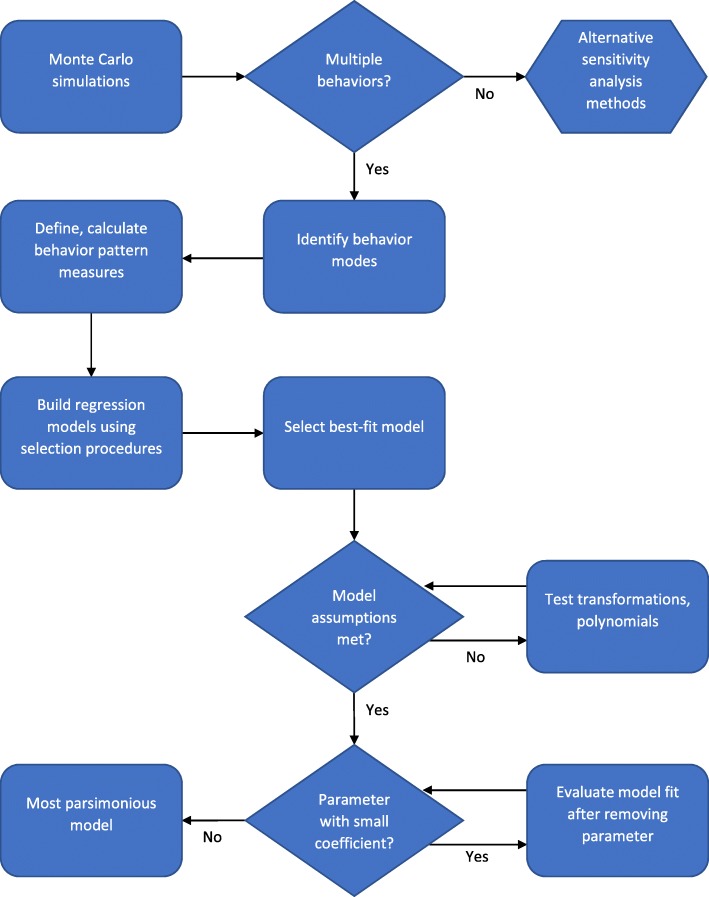
Behavioral sensitivity analysis process. The outputs of Monte Carlo simulations of mathematical models are classified into behavior pattern modes and pattern measures are defined for each of the modes. Standardized input parameter values from Monte Carlo simulations are used to build regression models for each pattern measure from each of the behavior pattern modes. Smoothing spline curves are fit to the simulation outputs if necessary to eliminate noise and enable calculation of the behavior pattern measures. Variable selection and model fit evaluation methods are used to find each best-fit regression model. Validity of assumptions for the best-fit regression model is evaluated; dependent (simulation outputs) and independent (parameters) variable transformations or other appropriate approaches such as time-dependent coefficients are used to meet the regression model assumptions if necessary. To obtain a most parsimonious regression model, parameters with relatively small coefficients in the best-fit model are eliminated, starting with the smallest, if there is no substantial change in model fit or the other parameter coefficients. Validity of assumptions is re-evaluated for the most parsimonious regression model

Behavior pattern sensitivity analysis has been proposed as an alternative and complement to standard sensitivity analysis when a model produces more than one mode of output behavior [20] because the behavior mode can confound associations between model parameters and outputs. We use the terms “behavior” and “behavior mode” to describe the model output pattern when the output is plotted over simulation time. Examples of behavior modes include, but are not limited to, oscillations, exponential decay, logarithmic growth, or sigmoid growth. Hekimoğlu et al. suggest a framework, similar to the first five steps of Fig. 1, for conducting and interpreting behavior pattern global sensitivity analysis on system dynamics models [20]. Behaviors are identified, characterized, and then regression models with standardized input parameter values are built to determine the association between input parameters and behavior pattern measures (Fig. 1) [20]. Such analysis can be useful for other model types that produce more than one behavior pattern or for situations where the output behavior matters more to the modeler than a single output value.

Here we apply behavior pattern sensitivity analysis for the first time to a pharmacokinetic-pharmacodynamic and bacterial ecology model of antimicrobial resistance in enteric bacteria in a beef steer during and after oral chlortetracycline administration. In 2011, the last time the U.S. national beef herd was surveyed about antimicrobial use, 71.7% of feedlots used chlortetracycline with 18.4% of all cattle receiving chlortetracycline in their feed during the feedlot period [21]. Most feedlots use chlortetracycline for disease prevention and control rather than disease treatment [21]. Although the U.S. Food and Drug Administration has recently prohibited in-feed use of medically important antimicrobials for growth promotion, such use for disease prevention, control and treatment is still permitted under the oversight of a veterinarian [22]. It is unclear whether this change will increase the use of chlortetracycline due to increased disease or decrease its use because of veterinarian oversight. Target pharmacologic models (empirical or physiologically based), with appropriate sensitivity analyses, can help create judicious antimicrobial-use policies by predicting antimicrobial concentrations in body compartments, such as the intestine, and evaluating alternative dosage regimens [7].

We build upon the original behavior pattern sensitivity analysis framework [20]. First, we incorporate principles and methods of model selection and model fit comparisons in order to improve parameter prioritization. By identifying which parameters contribute the most to model behavior variability, research efforts can be directed to produce data to reduce uncertainty in the values of those parameters. Next, we suggest techniques to address statistical assumption violations, including Cox proportional hazard models as an alternative to linear regression when linear regression assumptions are not met. Finally, we recommend building parsimonious models by removing parameters with small coefficients from the regression models in order to improve interpretability without sacrificing model fit. Using the example model of resistant enteric bacteria, we demonstrate how behavior pattern sensitivity analysis can be used for parameter prioritization and improved understanding of the modelled system.

## Results

The proportion of resistant enteric bacteria during and after chlortetracycline administration fits into one of three defined behaviors in all 1000 simulations: increasing, decreasing, or peaked. Sixty-seven simulations had increasing behavior of the proportion of resistant enteric *Escherichia coli* over time, 311 simulations had decreasing behavior, and 622 simulations had peaked behavior (an increase in proportion resistant during chlortetracycline administration followed by a decrease). We identified 3 behavior pattern measures that characterized these behaviors: equilibrium points, inflection points, and maximum points. These pattern measures were calculated in absolute terms (the proportion resistant at the time of occurrence) and in relative terms, in which the starting proportion resistant (Day 2) was subtracted from the proportion resistant at the time of occurrence. Specifically, increasing behavior was defined by absolute and relative equilibriums; decreasing behavior was defined by inflection points, and relative and maximum equilibriums; and peaked behavior was defined by absolute and relative maximum and equilibrium points. However, not every simulation achieved each behavior pattern measure during the simulated time period (90 days). The proportion resistant achieved equilibrium in only 36% of increasing behavior simulations by Day 90. Equilibrium was reached by 38% of decreasing behavior simulations and only 24% of peaked behavior simulations after chlortetracycline administration ended and before Day 90. Inflection points occurred during chlortetracycline administration in 65% of decreasing behavior simulations. For each behavior pattern measure and behavior mode, only simulations that had the behavior pattern measure were included in the linear regression models (i.e. missing data was removed pairwise). A maximum proportion resistant during chlortetracycline administration could be calculated for all peaked behavior simulations. In 80% of such simulations, the maximum occurred at the last time step during chlortetracycline administration because the removal of chlortetracycline inevitably caused a decline in proportion resistant for this behavior mode.

**Fig. 2:**
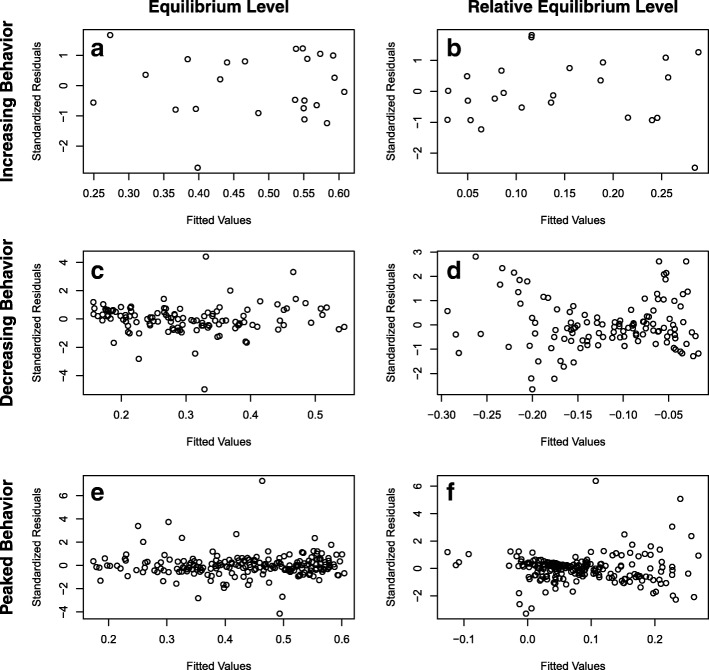
Standardized residuals of the proportion-resistant equilibrium level linear regression models for the three behavior modes. The three behaviors of the resistant bacteria in the example mathematical model were: (**a**, **b**) increasing, (**c**, **d**) decreasing, and (**e**, **f**) increasing during antimicrobial therapy and decreasing after therapy ceases (peaked). Separate linear regression models were built for each of the absolute (**a**, **c**, **e**) and relative (**b**, **d**, **f**) equilibrium levels in each behavior mode and are described in Table 1. The relative equilibrium level (**b**, **d**, **f**) is the proportion of resistance at the equilibrium point minus the starting proportion of resistance. The fitted values of the equilibrium level outcome are shown on the x-axis and the standardized residual values are shown on y-axis

The results from the final regression models for absolute and relative equilibrium levels of the proportion of resistant enteric bacteria are presented in Table 1. Five input parameters predominated in these models: *p*_*r*_ (proportion of resistance among bacteria flowing into the large intestine); *start*_*r*_ (Day 0 proportion of resistant bacteria in the large intestine), *λ*_*in*_ (rate of bacteria flowing into the large intestine, proportional to the total bacteria population size), *λ*_*out*_ (rate of bacteria flowing out of the large intestine, proportional to the total bacteria population size), *α* (fitness cost of resistance for the intermediate and resistant bacteria). The parameter *p*_*r*_ consistently had the largest coefficient in the regression models, indicating that a one standard deviation change in *p*_*r*_ had the largest effect on the equilibrium proportion resistant in the large intestine. The parameter *start*_*r*_ was significant in the relative equilibrium models only and it opposed the effect of *p*_*r*_. In general, the reduced regression models had improved fit (smaller AIC and BIC) over the full models (full models included all 26 input parameters of the mathematical model as independent variables), except for the regression models for the increasing behavior mode. The final reduced regression models for all the equilibrium pattern measures and all three behavior modes had high explanatory power (*R*^*2*^ ≥ 0.98). Residual plots from the absolute and relative equilibrium level models are shown in Fig. 2: the models of absolute equilibrium level met the assumption of homoscedasticity of residuals, while the relative equilibrium level models for decreasing and peaked behavior violated that assumption.

**Table 1:** Linear regression models for proportion-resistant absolute and relative equilibrium levels of the three behavior modes

Behavior Mode	Behavior Pattern Measure	Most Parsimonious Model								Full Model		
		Standardized Input Parameters Coefficient (Standard Error)					Fit Statistics			Fit Statistics		
		*p*_*r*_	*start*_*r*_	*λ*_*in*_	*λ*_*out*_	*α*	AIC	BIC	Adj. *R*^*2*^	AIC	BIC	Adj. *R*^*2*^
Increasing	Equilibrium Level	0.101 (0.0006)		0.005 (0.0007)		−0.004 (0.0007)	− 203	−197	.999	− 363*	− 335*	1*
Increasing	Relative Equilibrium Level	0.069 (0.002)	−0.068 (0.002)				− 147	−142	.986	− 239*	−211*	0.999*
Decreasing	Equilibrium Level	0.097 (0.0001)		0.001 (0.0002)	−0.001 (0.0001)	−0.001 (0.0001)	− 1321	− 1305	0.999	− 1309	− 1231	0.999
Decreasing	Relative Equilibrium Level	0.065 (0.0004)	−0.063 (0.0004)	0.013 (0.0008)			− 971	− 957	0.997	− 960	− 882	0.997
Peaked	Equilibrium Level ^A^	0.121 (0.0001)		0.003 (0.0002)	−0.001 (0.0001)	−0.002 (0.0001)	− 2040	− 2020	0.999	− 2030	− 1936	0.999
Peaked	Relative Equilibrium Level ^B^	0.081 (0.0005)	−0.081 (0.0005)	−0.007 (0.0007)			− 1566	− 1549	0.994	− 1562	− 1468	0.995

The example mathematical model was for the proportion of tetracycline-resistant enteric *Escherichia coli* in a beef steer during and after administration of oral chlortetracycline. A separate linear regression model was built for each behavior pattern measure of each behavior mode. The behavior pattern measure was the dependent variable in the linear regression models. Equilibrium was reached by 36% of increasing behavior simulations, 38% of decreasing behavior simulations and 24% of peaked behavior simulations after chlortetracycline administration ended and before the end of the simulation period. Simulations that did not reach equilibrium were excluded from these models. Coefficients and standard errors are listed for the standardized parameters that were included in each most parsimonious linear regression model. Full model refers to a linear regression model including all the parameters listed in Table 6 as independent variables. Akaike Information Criterion (AIC), Bayesian Information Criterion (BIC), and adjusted *R*^*2*^ are given for the most parsimonious and the full model. *Full model excludes log_10_(*β*_*ir*_), log_10_(*β*_*sr*_), *γ*_*s*_ and *MIC*_*i*_ to prevent overfitting. ^A^Peaked equilibrium level has 3 outliers removed (from reduced model and from full model). ^B^Peaked relative equilibrium level has one outlier removed (from reduced model and from full model)

A greater number of parameters were consequential in the final regression models for equilibrium time, compared to the equilibrium level models (Table 2). In addition, the parameters that were significant and consequential to model fit were different in each of the behavior modes, with the exception of *p*_*r*_ and *start*_*r*_ which were in the equilibrium time models for all three behavior modes. The coefficients for these and other parameters had similar absolute values, indicating that the parameters had approximately equal contributions to explaining variation in equilibrium time in all three behavior modes. In contrast, in the equilibrium level models, *p*_*r*_ often had a coefficient that was 5 to 100 times larger than other parameter coefficients (Table 1). The equilibrium time models had lower explanatory power than the equilibrium level models (*R*^*2*^ < 0.74) (Table 2 vs. Table 1).

**Table 2:** Linear regression models for time to proportion-resistant equilibrium of the three behavior modes

Behavior Mode	Behavior Pattern Measure	Most Parsimonious Model														Full Model		
		Standardized Input Parameters Coefficient (Standard Error)											Fit Statistics			Fit Statistics		
		*p*_*r*_		*start*_*r*_	*λ*_*in*_	*λ*_*out*_	*δ*	*MIC*_*s*_	*H*_*i*_	*V*_*LI*_	*η*_*LI*_	*γ*_*LI*_	AIC	BIC	Adj *R*^*2*^	AIC	BIC	Adj *R*^*2*^
Increasing	Equilibrium time	1868 (347)		− 883 (313)		− 1085 (352)			− 1526 (352)	− 884 (321)		− 1141 (292)	427	437	0.733	436*	464*	−0.706*
Decreasing	Equilibrium Time	*p*_*r*_	− 1527 (514)	1233 (207)	− 3488 (396)		− 750 (175)	− 1107 (180)		− 664 (179)			2141	2172	0.561	2149	2226	0.583
		*p*_*r*_^2^	805 (408)															
		*p*_*r*_^3^	552 (331)															
		*p*_*r*_^4^	− 410 (156)															
Peaked	Equilibrium Time	810 (175)		− 667 (174)	− 1807 (279)	660 (139)	− 781 (123)	− 1393 (154)		− 842 (145)	− 628 (146)		3812	3846	0.423	3824	3918	0.436

The example mathematical model was for the proportion of tetracycline-resistant enteric *Escherichia coli* in a beef steer during and after administration of oral chlortetracycline. A separate linear regression model was built for each behavior pattern measure of each behavior mode. The behavior pattern measure was the dependent variable in the linear regression models. Equilibrium was reached by 36% of increasing behavior simulations, 38% of decreasing behavior simulations and 24% of peaked behavior simulations after chlortetracycline administration ended and before the end of the simulation period. Simulations that did not reach equilibrium were excluded from these models. Coefficients and standard errors are listed for the standardized parameters that were included in each most parsimonious linear regression model. Full model refers to a linear regression model including all the parameters listed in Table 6 as independent variables. Akaike Information Criterion (AIC), Bayesian Information Criterion (BIC), and adjusted *R*^*2*^ are given for the most parsimonious and the full model. *Excludes log_10_(*β*_*ir*_), log_10_(*β*_*sr*_), *γ*_*s*_ and *MIC*_*i*_ to prevent overfitting

**Fig. 3:**
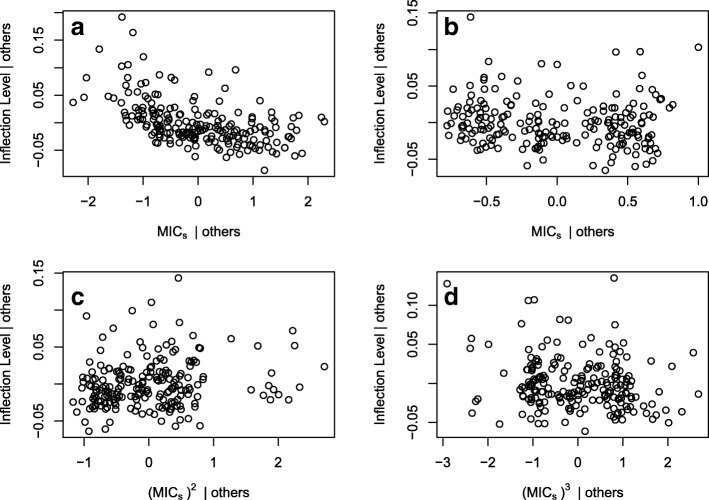
Partial regression plots from the proportion-resistant inflection level linear regression models in decreasing behavior simulations. The example mathematical model was for the proportion of tetracycline-resistant enteric *Escherichia coli* in a beef steer during and after administration of oral chlortetracycline. **a** is a partial regression plot for a regression model that contains no polynomial terms and shows the effect of *MIC*_*s*_ on the inflection level of resistance after accounting for all other variables in the model. **b**-**d** are partial regression plots for a regression model that contains polynomial terms of *MIC*_*s*_ and show the effects of (**b**) *MIC*_*s*_, (**c**) *MIC*_*s*_^*2*^, and (**d**) *MIC*_*s*_^*3*^ after accounting for all other variables in the polynomial model

Inflection points only occurred in the decreasing behavior mode and they occurred during chlortetracycline administration (between Day 2 and Day 30). Parameters related to the bacterial population dynamics and chlortetracycline pharmacokinetics-pharmacodynamics all made significant contributions to predicting the proportion of resistance at the inflection point (Table 3). In contrast, only parameters of the bacterial population dynamics were significantly associated with the proportion of resistance at equilibrium points. Consistent with the equilibrium level models, *p*_*r*_ had the largest coefficient in the inflection level model (Tables 1 and 3). The pharmacokinetic parameters *δ* (chlortetracycline abiotic degradation rate), *V*_*LI*_ (volume of large intestine), and *η*_*LI*_ (fraction of chlortetracycline adsorbed to digesta) all had small to moderate negative coefficients. Polynomial terms of *MIC*_*s*_ (susceptible *E. coli* MIC) were added to improve the linearity between *MIC*_*s*_ and inflection proportion of resistance (Fig. 3). The addition of polynomial terms resulted in a minor improvement in model fit compared to a model without polynomial terms (R^2^ = 0.885, AIC = − 800, BIC = − 767 with polynomial terms compared to R^2^ = 0.864, AIC = − 767, BIC = − 741 without). The final inflection level model explained a large amount of variation in the proportion of resistant *E. coli* at the inflection point (*R*^*2*^ = 0.885).

**Table 3:** Linear regression models for proportion-resistant inflection and maximum levels of decreasing and peaked behaviors, respectively

Behavior Mode	Behavior Pattern Measure	Most Parsimonious Model													Full Model		
		Standardized Input Parameters Coefficient (Standard Error)										Fit Statistics			Fit Statistics		
		*p*_*r*_	*start*_*r*_	*λ*_*in*_	*λ*_*out*_	log_10_*(β*_*sr*_*)*	*MIC*_*s*_		*δ*	*V*_*LI*_	*η*_*LI*_	AIC	BIC	Adj. *R*^*2*^	AIC	BIC	Adj. *R*^*2*^
Decreasing	Inflection Level	0.091 (0.002)		−0.031 (0.003)			*MIC*_*s*_	−0.007 (0.005)	−0.02 (0.004)	− 0.011 (0.002)	− 0.011 (0.002)	−800	−767	0.885	− 758	−664	0.869
							*MIC*_*s*_^*2*^	0.008 (0.003)									
							*MIC*_*s*_^*3*^	−0.006 (0.002)									
Peaked	Max Level	0.086 (0.003)	0.022 (0.003)	−0.036 (0.003)	0.02 (0.002)	0.009 (0.002)	*MIC*_*s*_	−0.081 (0.003)	−0.021 (0.002)	− 0.022 (0.002)	−0.018 (0.002)	− 1706	− 1653	0.807	− 1597	−1468	0.775
							*MIC*_*s*_^*2*^	0.032 (0.003)									
Peaked	Relative Max Level	0.06 (0.003)	−0.07 (0.003)	−0.043 (0.003)	0.02 (0.003)		−0.02 (0.003)		−0.068 (0.003)	− 0.022 (0.003)	−0.021 (0.003)	− 1685	− 1641	0.734	− 1709	− 1585	0.752

The example mathematical model was for the proportion of tetracycline-resistant enteric *Escherichia coli* in a beef steer during and after administration of oral chlortetracycline. A separate linear regression model was built for each behavior pattern measure of each behavior mode. The behavior pattern measure was the dependent variable in the linear regression models. Inflection points occurred during chlortetracycline administration in 65% of decreasing behavior simulations. Simulations that did not have an inflection point were excluded from the inflection level model. A maximum proportion resistant during chlortetracycline administration could be calculated for all peaked behavior simulations. Coefficients and standard errors are listed for the standardized parameters that were included in each most parsimonious linear regression model. Full model refers to a linear regression model including all the parameters listed in Table 6 as independent variables. Akaike Information Criterion (AIC), Bayesian Information Criterion (BIC), and adjusted *R*^*2*^ are given for the most parsimonious and the full model

**Fig. 4:**
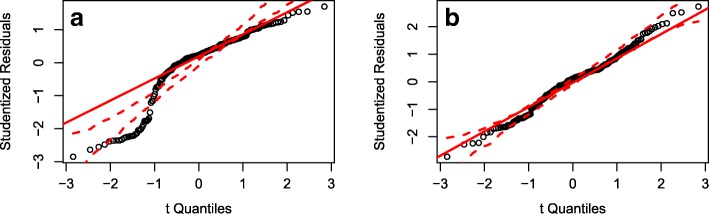
Quantile-quantile plots for the decreasing behavior inflection time and inflection time to the fourth power. Residuals from a linear regression model with (**a**) inflection time as the output and (**b**) inflection time to the fourth power as the output are plotted on the y-axis. The theoretical quantiles of a normal distribution are plotted on the x-axis. The solid red line passes through the quartile-pairs and the dotted red lines encompass a 95% confidence interval for the theoretical normal distribution

The linear regression model for the inflection time in decreasing behavior simulations violated the assumption of normally distributed residuals but the residual distribution was significantly improved by modeling inflection time to the fourth power (Fig. 4). Only three parameters had a substantial impact on the inflection time: *p*_*r*_, *start*_*r*_, and *MIC*_*s*_. The proportion of resistance in inflowing bacteria (*p*_*r*_) and the starting proportion of resistance (*start*_*r*_) had approximately equal but opposing effects (Table 4). A higher inflowing proportion of resistance shortened the time to the inflection point whereas a higher starting level of resistance increased the time to the inflection point. A higher chlortetracycline MIC for the susceptible bacteria also increased the time to the inflection point. The reduction from 26 to three parameters did not significantly affect the model fit, although the full and reduced models both had low explanatory power (*R*^*2*^ = 0.3 for the reduced model).

**Table 4:** Linear regression models for proportion-resistant inflection and maximum times of decreasing and peaked behaviors, respectively

Behavior Mode	Behavior Pattern Measure	Most Parsimonious Model										Full Model		
		Standardized Input Parameters Coefficient (Standard Error)							Fit Statistics			Fit Statistics		
		*p*_*r*_	*p*_*i*_	*start*_*r*_	*start*_*i*_	*λ*_*out*_	*MIC*_*s*_	*η*_*LI*_	AIC	BIC	Adj. *R*^*2*^	AIC	BIC	Adj. *R*^*2*^
Decreasing	Inflection Time^4^	−3.2e14 (4.5e13)		3.4e14 (4.6e13)			2.2e14 (4e13)		14,373	14,389	0.301	14,367	14,460	0.387
Peaked	Max Time	310.43 (31.71)	−226.84 (30.33)	− 282.98 (31.6)	115.5 (30.3)	− 102.45 (30.31)	270.5 (31.02)	61.23 (30.63)	10,014	10,054	0.348	9853	9982	0.512

The example mathematical model was for the proportion of tetracycline-resistant enteric *Escherichia coli* in a beef steer during and after administration of oral chlortetracycline. A separate linear regression model was built for each behavior pattern measure of each behavior mode. The behavior pattern measure was the dependent variable in the linear regression models. Inflection points occurred during chlortetracycline administration in 65% of decreasing behavior simulations. Simulations that did not have an inflection point were excluded from the inflection level model. A maximum proportion resistant during chlortetracycline administration could be calculated for all peaked behavior simulations. Coefficients and standard errors are listed for the standardized parameters that were included in each most parsimonious linear regression model. For the inflection time model, the dependent variable was inflection time raised to the fourth power. Full model refers to a linear regression model including all the parameters listed in Table 6 as independent variables. Akaike Information Criterion (AIC), Bayesian Information Criterion (BIC), and adjusted *R*^*2*^ are given for the most parsimonious and the full model

Similar to the inflection level model for the decreasing behavior, the maximum level models for the peaked behavior incorporated parameters for the pharmacokinetics of chlortetracycline, *E. coli* population, and the pharmacodynamics (Table 3). In addition, the fit also improved from the full to the reduced model for absolute inflection level and the absolute maximum level. All the parameters from the absolute inflection level model were also included in the absolute and relative maximum level models and the coefficients had similar magnitude and direction (Table 3). The maximum level models also included effects for the starting proportion of resistance, outflow rate of bacteria, and one plasmid transfer term (absolute maximum level model only). The starting level of resistance had opposite effects on the absolute and relative maximum levels of resistance. An increase in the starting level of resistance had a positive association with the absolute maximum level of resistance but a negative association with the relative maximum level of resistance, reflecting the calculation of the relative level as the absolute minus the starting level.

**Fig. 5:**
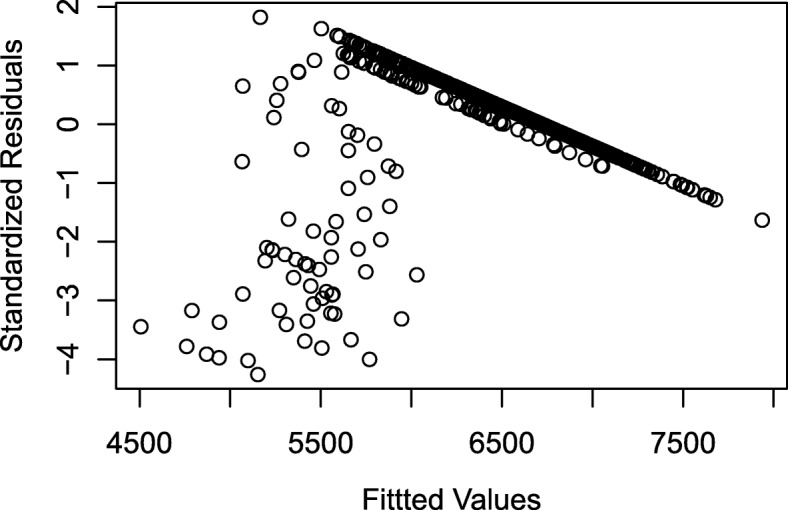
Standardized residuals of the time of maximum proportion resistant regression model for the peaked behavior. The fitted values of the time of maximum outcome are shown on the x-axis and the standardized residual values are shown on y-axis

The time of the maximum proportion resistant in peaked behavior simulations exhibited large violations of linear model assumptions (Fig. 5) and therefore should not be interpreted. This occurred because 80% of the simulations had a maximum proportion resistant at the last time-point of chlortetracycline administration, indicating that they had not reached an absolute maximum but instead had a local maximum due to the abrupt change in chlortetracycline input. Therefore, a Cox proportional hazard model was fit for the time of maximum resistance (‘time to’ the ‘event’ of maximum resistance). A non-censoring model was developed that considered all simulations to have reached a maximum, i.e. considering as the event a local or absolute maximum. In a second censored model, only reaching an absolute maximum was considered as the event; those simulations that had increasing resistance at the end of chlortetracycline administration and may have reached an absolute maximum at a later time-point were censored. The parameters retained in the best, most-parsimonious non-censored and censored models were the same, with moderate changes in parameter coefficients but no change in coefficient signs between the two models. However, several parameters in the best model (censored) violated the proportional hazard assumption: the proportion of resistant inflowing bacteria, the proportion of intermediate inflowing bacteria, the starting proportion of resistance, the inflow rate of bacteria, and the MIC of susceptible bacteria. The right-censored model was used to address the proportional hazards violation. A continuous time-dependent coefficient function could not be identified for these five parameters, therefore a step-function was used to create proportional hazards [23]. The range of maximum times was divided into equal thirds: Day 10 to Day 16.6, Day 16.7 to Day 23.3, and Day 23.4 to Day 30. A right-censored Cox proportional model was then fit with this step function for the five non-proportional hazard parameters. The resulting model coefficients are presented in Table 5.

**Table 5:** Cox proportional hazard model for time of maximum proportion resistant of the peaked behavior mode

Standardized Input Parameter	Time Strata	Coefficient	Exponentiated Coefficient	Standard Error
*p*_*r*_	1	−2.133	0.118	0.33
	2	−1.274	0.280	0.30
	3	−1.199	0.302	0.166
*p*_*i*_	1	2.156	8.639	0.311
	2	1.748	5.745	0.305
	3	0.691	1.996	0.136
*start*_*r*_	1	1.945	6.996	0.321
	2	1.249	3.485	0.291
	3	1.299	3.666	0.164
*start*_*i*_		−0.856	0.425	0.110
*λ*_*in*_	1	0.810	2.248	0.245
	2*	0.164	1.179	0.250
	3	1.038	2.822	0.143
*λ*_*out*_		0.581	1.787	0.099
log_10_(*β*_*sr*_)		0.206	1.228	0.098
MIC_s_	1	−8.913	0.0001	1.474
	2	−3.631	0.026	0.763
	3	−0.824	0.439	0.159
*δ*		−0.343	0.710	0.131
*V*_*LI*_		−0.481	0.618	0.113
*η*_*LI*_		−0.384	0.681	0.096

The example mathematical model was for the proportion of tetracycline-resistant enteric *Escherichia coli* in a beef steer during and after administration of oral chlortetracycline. The Cox model presented here uses right-censored data. In 80% of peaked behavior simulations the maximum proportion resistant occurred at the last time step of chlortetracycline administration and these simulations were considered to not have a maximum event occur in the Cox model (right-censored). Coefficients, exponentiated coefficients, and standard errors are listed for the standardized parameters that were included in the most parsimonious model. Time stratification, which allows the effect of a parameter to vary as a step-function between time strata, was used to meet the assumption of proportional hazards for *p*_*r*_, *p*_*i*_, *start*_*r*_, *λ*_*in*_, and *MIC*_*s*_. The maximum times were divided into three time strata: (1) Day 10 to Day 16.6, (2) Day 16.7 to Day 23.3, and (3) Day 23.4 to Day 30. Coefficients for these five parameters were constant within a time stratum and different between time strata. Parameters that do not have time strata listed met the proportional hazard assumption without stratification and have just one coefficient, constant across time. For this model, Cox and Snell’s *R*^*2*^ was 0.248 (maximum possible 0.584). *P > 0.05

With the step function, all parameters met the assumption for proportional hazards. All three pharmacokinetic parameters (chlortetracycline abiotic degradation rate, large intestine volume, and adsorption of chlortetracycline to digesta) included in the maximum time Cox model had negative coefficients, indicating that an increase in those parameters is associated with a decrease in the hazard of reaching a maximum resistant proportion. For example, the hazard decreases by 29% when the degradation rate (*δ*) increases by one standard deviation. A decrease in hazard corresponds to a lower probability of reaching a maximum and hence a greater time to maximum. On the other hand, an increase in hazard corresponds to a greater probability of reaching maximum and a shorter time to maximum. An increase in the pharmacodynamic parameter *MIC*_*s*_ is also associated with a decrease in hazard but this association changes over the time strata. The *MIC*_*s*_ has a larger impact on hazard early in the chlortetracycline administration period, compared to later in the time period. This change over time was also true for *p*_*r*_, although it had a more modest impact on the hazard of reaching maximum. An increase in *p*_*i*_ and *start*_*r*_ both had a positive association with the hazard and a stronger effect at the beginning of the time period. Increases in both *λ*_*in*_ and *λ*_*out*_ also had positive associations with the hazard and the coefficient for *λ*_*in*_ changed over time.

## Discussion

Behavior pattern sensitivity analysis can be useful for models that have multiple output behaviors because it includes separate behavior pattern measures and statistical analyses for each output behavior mode. In general, output behavior modes can be simple or complex, few or numerous [20]. Correctly classifying the behavior mode of each simulation is important to the validity of the analysis and misclassification could bias the regression models. At best, if the different behavior modes have similar associations with the same input parameters (as in Table 1), then there may be little to no discernable impact of behavior misclassification. However, if the different behavior modes are associated with different input parameters or have opposite associations with the same parameters (as in Table 2), then behavior misclassification can lead to bias towards the null and the effect of input parameters may be missed. Since the three behavior modes in the example mathematical model could be distinguished based on three timepoints (Day 2, Day 30, Day 90) and all simulations could be classified as one of the three behaviors, there was likely little to no misclassification in the example sensitivity analysis.

By applying model selection methods (e.g., stepwise variable selection) and model fit measures (*R*^*2*^, BIC, AIC) to behavior pattern sensitivity analysis we have demonstrated how a large number of model input parameters can be efficiently prioritized. Appropriate parameter prioritization helps to focus research efforts on parameters that have the largest impact on the model output. Even though many parameters were statistically significant after the initial regression model selection, parameters that do not explain a large amount of variability in the outcome are likely not useful in manipulating real-world systems. By using standardized parameter values, the absolute values of the regression coefficients can be used to prioritize parameters for each behavior mode and behavior pattern measure [20]. Creating a parsimonious model by removing parameters with small coefficients (< 0.005 in the “level” models, < 240 in the “time” models), without sacrificing model fit or affecting other parameter coefficients, facilitates interpretation of sensitivity analyses. This is similar to the motivation behind LASSO regression, which produces more interpretable linear models by constraining the sum of the coefficients [24].

Large associations between input parameters and behavior pattern measures can indicate that the parameters may have important roles in the biological system and are leverage points for manipulating the system [2, 20]. On the other hand, caution should be exercised because a large association could be due to inaccuracies in model structure or substantial uncertainty in the parameter estimate [17, 18]. In some modeling situations, sensitivity analyses are useful for identifying uninfluential parameters so they can be fixed at a best-estimate value to reduce model complexity [17]. Since we sought to build the smallest possible linear regression models without sacrificing model fit, we cannot directly evaluate which parameters had the smallest impact on the behavior pattern measures. When using regression modeling for behavior pattern sensitivity analysis, this goal is best achieved by examining the full regression models [20].

In our example, behavior pattern sensitivity analysis of a mathematical model of enteric antimicrobial resistance in beef cattle fed chlortetracycline, parsimonious regression model selection identified parameters that had a significant and substantial effect on the proportion of resistant bacteria during and after chlortetracycline administration. Some parameters (*p*_*r*_, *start*_*r*_, *λ*_*in*_, *λ*_*out*_, *MIC*_*s*_, *δ*, *V*_*LI*_, *η*_*LI*_) were associated with several or all the resistant proportion behavior pattern measures and are therefore considered to have great importance in the model of antimicrobial resistance. Most of these parameters were also previously identified via Spearman correlation as associated with the average proportion of enteric resistance during chlortetracycline administration, across all simulations and behaviors [11]. The proportion of inflowing resistance (*p*_*r*_) consistently had a coefficient five to ten times larger than other parameters when predicting the level of enteric resistance (Tables 1, 3). Therefore, this variable could be useful as an intervention to alter the level of enteric resistance. Many parameters have similarly large coefficients in the equilibrium time models (Table 2) and inflection time model (Table 4). Pharmacokinetic and pharmacodynamic parameters had a significant impact on the level of resistance during (Table 3) but not after (Table 1) chlortetracycline administration. Therefore, depending on the outcome and time of interest in the modelled system, it may or may not be worthwhile to expend effort to reduce the uncertainty in the pharmacokinetic and pharmacodynamic parameters. The behavior pattern sensitivity analysis also identified parameters (*α*, *γ*_*LI*_, log_10_(*βsr*), *H*_*i*_) that were associated with only one or a few behavior pattern measures and were not identified as significant in the previous sensitivity analysis for the average resistant proportion during chlortetracycline administration [11]. These variables could be ranked as ‘second-tier’ importance during parameter prioritization.

In many practical modeling problems, it is important to have a detailed sensitivity analysis to translate model findings to real-world applications. For example, all three behavior modes had the proportion resistant at equilibrium affected by the same input parameters with similar coefficients (Table 1). Therefore, we can be confident that changes to those input parameters in the real-world will have consistent effects, despite individual variation in enteric resistance behavior. If the level of resistance after chlortetracycline treatment matters to beef producers and public health officials, then they can focus on changing the variables with the strongest, consistent associations with the equilibrium level of resistance. Diet changes could be used to reduce the proportion of inflowing resistant bacteria (*p*_*r*_) and the rate of the inflow (*λ*_*in*_) and thereby reduce the equilibrium level of resistant enteric bacteria. For example, added probiotics [25, 26] and silage-based diets [27] have been evaluated for such purposes. However, such an intervention may have complex effects on the level of enteric resistance during chlortetracycline treatment, when *p*_*r*_ has a positive association with resistance levels but *λ*_*in*_ has a negative association (Table 3). The effect of a diet change on the time required to reach the post-chlortetracycline equilibrium (effectively a resistance-based withdrawal period) may differ by the underlying behavior of enteric bacteria in individual animals (Table 2).

We used polynomial terms to address non-linear relationships between model input parameters and the behavior pattern measures, although polynomial terms can be difficult to interpret. For example, in the maximum proportion resistant linear regression model for the peaked behavior, the coefficient of *MIC*_*s*_ is negative and the coefficient of *MIC*_*s*_^*2*^ is positive. The combined effect is negative when *MIC*_*s*_ is between 0 and 2.5 standard deviations above its mean and positive when *MIC*_*s*_ is below its mean or greater than 2.5 standard deviations above its mean. We suggest plotting the polynomial equations to aid in interpretation; then the combined effect of the linear and polynomial terms at a given input parameter value can be compared relative to other input parameter coefficients in the linear regression model. Although using polynomials can correct violations of linear model assumptions, they may not substantially increase the model fit and therefore may not be worth the complexity of interpretation. We did not pursue interactions between input parameters because they can be difficult to interpret, particularly 3^rd^ order and higher interactions. However, input parameters may interact in some model structures and therefore interactions may need to be included in regression models for the sensitivity analysis.

In their behavior pattern sensitivity analysis framework, Hekimoğlu et al. [20] do not address censored data, although censoring can occur in mathematical models when an event does not occur during the simulated time period. In our example model, some simulations did not reach an equilibrium point in the simulated time period, although all simulations tended towards an equilibrium and would presumably reach an equilibrium if the simulation time was extended. There are three potential solutions to this problem: (1) extend the simulation time until all simulations have the required event, (2) remove missing data pairwise, or (3) use statistical methods for censored data in the sensitivity analysis. Solution (1) may not reflect the reality of animal production systems. Animals are sent to slaughter at predetermined times and cannot necessarily be held at farms until their gut microbiota have equilibrized following removal of antimicrobial therapy. In addition, there are practical and legal limitations on how long antimicrobials can be fed to production animals [28], so the chlortetracycline administration time cannot be extended until all simulated animals reach an equilibrium or maximum during the drug administration. In most of linear regression models presented, we used strategy (2), but for the time to maximum in peaked behavior simulations we applied strategy (3) because the linear regression model severely violated the assumption of normally distributed residuals. We used survival analysis (Cox proportional hazard model) to account for ‘censored’ simulations—those that did not reach a maximum proportion resistant during chlortetracycline administration but rather had a recorded maximum at the last time-point of the administration. Although Cox proportional hazard models have fewer assumptions than linear regression models, the example model data violated the important assumption of proportional hazards, which was addressed by using a step-function (time dependent coefficients) within a right-censored model. The most parsimonious Cox model (Table 5) contained more input parameters than the most parsimonious linear regression model for the time to maximum (Table 4); the Cox model identified all the parameters in the linear regression model (Table 4) plus four additional parameters (Table 5), supporting its suitability for the purpose.

## Conclusions

Behavior pattern sensitivity analysis is a flexible method that can be applied to models of bacterial antimicrobial resistance, including antimicrobial pharmacokinetic-pharmacodynamic and bacterial population dynamics models. It provides a detailed sensitivity analysis for each model output behavior and highlights similarities and differences in parameter importance among the behaviors. By using stepwise and best subsets model selection techniques, we have expanded the procedures for behavior pattern sensitivity analysis to efficiently identify the parameters that have the strongest association with each behavior pattern measure. We suggest techniques for addressing violations of linear regression models, including transformations of dependent and independent variables and alternative models (Cox proportional hazard models), thus expanding the techniques for behavior pattern sensitivity analysis. Finally, in the example model of enteric antimicrobial resistance in beef cattle administered an oral antimicrobial, we demonstrate that behavior pattern sensitivity analysis identifies important parameters that could be altered to reduce antimicrobial resistance.

## Methods

### Example mathematical model

**Fig. 6:**
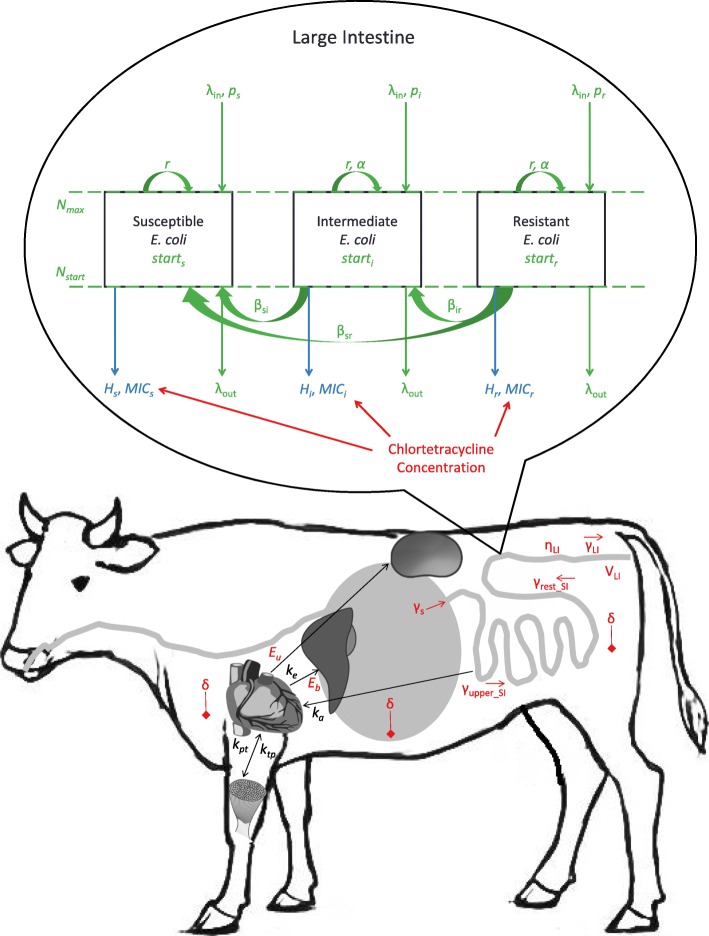
Schematic of the example mathematical model of tetracycline-resistant *Escherichia coli* in beef cattle administered oral chlortetracycline. Pharmacokinetic parameters related to the distribution of chlortetracycline throughout the gastrointestinal tract and excretion via urine and bile are presented in red. The black parameters are pharmacokinetic constants and were not varied during simulations: absorption rates from the small intestine (*k*_*a*_), excretion rates (*k*_*e*_) and distribution to (*k*_*pt*_) and from (*k*_*tp*_) tissues. Pharmacodynamic parameters related to the effect of the large intestine chlortetracycline concentration on enteric *E. coli* are given in blue. Parameters related to the bacterial population dynamics are given in green. The definition and distribution of each parameter are presented in Table 6

The mathematical model we use as an example has previously been described in detail [11] and is represented schematically in Fig. 6. In short, the model combines a pharmacokinetic stock-flow model of chlortetracycline in the beef cattle gastrointestinal tract [5] with a population dynamics model of resistant and susceptible enteric *Escherichia coli* and a pharmacodynamic (sigmoid *E*_*max*_) equation of the antimicrobial effect on the bacteria. For the application of behavior pattern sensitivity analysis we focused on one chlortetracycline indication and dosage: control of bacterial pneumonia in beef steers with chlortetracycline dosed at 350 mg/steer per day for 28 days. This dosage was chosen because disease prevention is the most common use of in-feed chlortetracycline in feedlot beef cattle [21]. The model reflected chlortetracycline flows between gastrointestinal, plasma, and tissue compartments and abiotic degradation. Within the large intestine, active chlortetracycline inhibited the growth of *E. coli* depending on their susceptibility; susceptible *E. coli* had slower growth in the presence of sub-inhibitory chlortetracycline concentrations than resistant *E. coli*. The model also reflected that resistance genes could be horizontally transferred between resistant, intermediate, and susceptible *E. coli*, and the bacterial population could be replenished by ingested *E. coli*. Twenty-nine input parameters were assigned distributions based on published literature (Table 6), including parameters for chlortetracycline pharmacokinetics, chlortetracycline pharmacodynamics against *E. coli*, and *E. coli* ecology. Monte Carlo simulations (*n* = 1000) of the model were completed by randomly drawing values from the parameter distributions for each simulation [11]; the random values were recorded as a realization of each input parameter random variable. The model was simulated with a 0.1 h time-step for a total of 90 days: a two day burn-in period, 28 days of chlortetracycline administration, and an additional 60 days of follow-up after stopping chlortetracycline. The model output was the proportion of chlortetracycline-resistant *E. coli* out of total enteric *E. coli* over time. The model was built and simulated in MatLab ® R2016b (MathWorks, Natick, MA, U.S.).

**Table 6:** Parameters, and their distributions, of the example mathematical model

Parameter	Distribution	Definition
CTC pharmacokinetics		
*δ*	Beta (0.54, 37.4)	CTC abiotic degradation rate
*γ*_*s*_	Uniform (0.0535, 0.0895)	CTC flow rate from stomachs to small intestine
*γ*_*upper_si*_	Uniform (0.250, 0.416)	CTC flow rate through the upper 1/3 small intestine
*γ*_*rest_si*_	Uniform (0.100, 0.166)	CTC flow rate through the lower 2/3 small intestine
*γ*_*LI*_	Uniform (0.100, 0.166)	CTC flow rate through large intestine
*E*_*b*_	Uniform (0.39, 0.64)	Fraction CTC eliminated via bile
*η*_*LI*_	Uniform (0.69, 0.89)	Fraction CTC adsorbed to digesta in the large intestine
*V*_*LI*_	Uniform (6, 22)	Large intestine contents volume
CTC pharmacodynamics		
*H*_*s*_	Uniform (1.62, 2.23)	Hill coefficient for susceptible bacteria
*H*_*i*_	Uniform (5.71, 9.53)	Hill coefficient for intermediate bacteria
*H*_*r*_	Uniform (6.42, 10)	Hill coefficient for resistant bacteria
*MIC*_*s*_	Uniform (0, 4)	Anaerobic MIC for susceptible bacteria
*MIC*_*i*_	Uniform (2.7, 16)	Anaerobic MIC for intermediate bacteria
*MIC*_*r*_	Uniform (14.7, 128)	Anaerobic MIC for resistant bacteria
Bacterial population dynamics in the large intestine		
*r*	Uniform (0.05, 0.5)	Bacterial growth rate in the large intestine
*α*	Uniform (0, 0.03)	Fitness cost for intermediate and resistant bacteria
log_10_(*N*_*max*_)	Weibull (14.03, 20.32) − 7.59	Large intestine carrying capacity for the bacteria
*N*_*start*_	Uniform (0.1, 0.9) * *N*_*max*_	Starting bacterial population size
log_10_(*β*_*jk*_)	Gamma (94.17,0.16) − 22.57	transposon transfer rate between /transposon transfer rate between *E. coli* subpopulations
*λ*_*in*_	Uniform (0.001, 0.01)	Bacterial in-flow rate to the large intestine
*λ*_*out*_	Uniform (0.01, 0.02)	Bacterial out-flow rate from the large intestine
*p*_*i*_	Uniform (0.02, 0.15)	Proportion intermediate in in-flowing bacteria
*p*_*r*_	Uniform (0.16, 0.61)	Proportion resistant in in-flowing bacteria
*p*_*s*_	1- *p*_*i*_ - *p*_*r*_	Proportion susceptible in in-flowing bacteria
*start*_*j*_	Same as *p*_*j*_	Starting proportions of resistant (*start*_*r*_), intermediate (*start*_*i*_), and susceptible (*start*_*s*_) bacteria in the large intestine

The model was for the proportion of tetracycline-resistant enteric *Escherichia coli* in a beef steer during and after administration of oral chlortetracycline, and has been previously published (see text). The parameter symbols, definitions, and distributions are given here. Chlortetracycline (CTC)

### Behavior pattern sensitivity analysis framework

The general process for behavior patterns sensitivity analysis is detailed in Fig. 1 and described below. We followed the framework laid out by Hekimoğlu et al. [20] for a behavior pattern sensitivity analysis on system dynamics models:

1. Run Monte Carlo simulations with predetermined parameter distributions
2. Identify and separate different output behavior modes
3. Define and compute output behavior pattern measures for every behavior mode
4. Perform regression analyses with output behavior pattern measures as dependent variables and standardized input parameter values as independent variables

### Behavior mode identification and separation

**Fig. 7:**
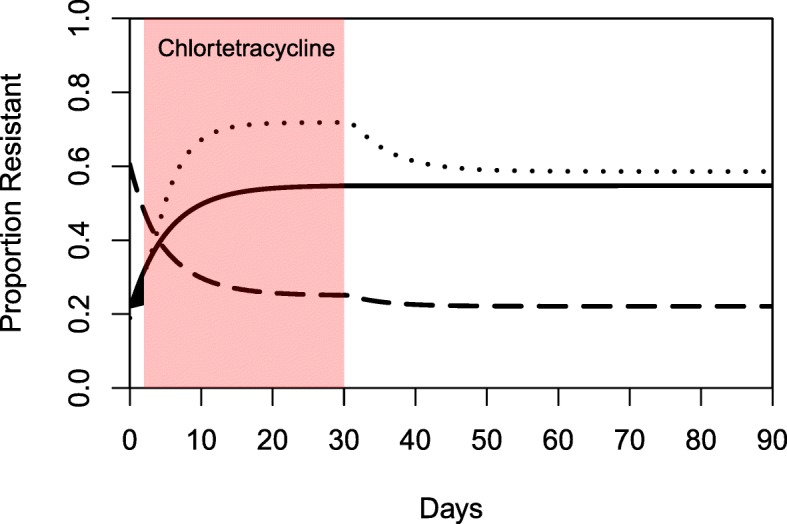
Examples of the behaviors of tetracycline-resistant enteric *Escherichia coli* in beef cattle administered oral chlortetracycline. The day of the simulation is shown on the x-axis and the proportion of tetracycline-resistant enteric *Escherichia coli* is shown on the y-axis. The red shaded box is the period of chlortetracycline administration from Day 2 to Day 30. The solid line is an example of the increasing behavior, with the proportion of resistance at Day 2 < Day 30 < Day 90. The dashed line is an example of the decreasing behavior, with the proportion of resistance at Day 2 > Day 30 > Day 90. The dotted line is an example of the peaked behavior, with the proportion of resistance at Day 2 < Day 30 > Day 90

After running 1000 Monte Carlo simulations, we examined the model output behaviors by plotting the proportion of tetracycline-resistant enteric bacteria over time. We visually examined all 1000 simulations by plotting the outputs of 100 simulations at a time and noting behavior trends. Three distinct behaviors were identified and confirmed by examining the proportion of resistance over time from a subset of simulations (Fig. 7): (1) an increase in the proportion of resistant *E. coli* across the entire 90 days, (2) a decrease in the proportion resistant across the entire 90 days, and (3) an increase in the proportion resistant during chlortetracycline administration followed by a decrease after stopping chlortetracycline. These behavior modes were formally defined by comparing the proportion resistant at the start of chlortetracycline administration (Day 2), at the end of administration (Day 30), and at the end of the simulation period (Day 90). Increasing behavior was defined as the proportion resistant at Day 2 < Day 30 < Day 90. Decreasing behavior was defined as the proportion resistant at Day 2 > Day 30 > Day 90. The third behavior was termed “peaked” and was defined as the proportion resistant at Day 2 < Day 30 > Day 90.

### Behavior pattern measures

Behavior pattern measures become the dependent variables in the regression analyses, with a separate regression model for each behavior pattern measure from each behavior mode. Hekimoğlu et al. suggest that the modeler should identify a set of behavior pattern measures that completely characterize each behavior mode. Suggested pattern measures included the output levels and timing of the equilibriums, inflection points, tipping points (peaks), as well as the oscillation periods and oscillation amplitude slopes [20]. The three behaviors observed in our model were characterized by the output equilibrium level, time to equilibrium, the output inflection point level, inflection point time, the output maximum level, and time of maximum. Maximums were found for the peaked behavior simulations by using the *max* MatLab function during the period of chlortetracycline administration (Day 2 to Day 30). We searched for equilibriums after ending chlortetracycline (> Day 30) and for inflection points during chlortetracycline administration (Day 2 to Day 30). Equilibriums and inflection points can be calculated based on the first and second derivatives of a curve, respectively. However, the proportion of resistant *E. coli* contained too much noise to calculate informative derivatives or gradients because chlortetracycline was fed in 12 h intervals, which created small oscillations in the *E. coli* proportions. Therefore, we first fit a smoothing spline curve with the least-squares method (using the *spaps* MatLab function) to each simulation’s output with a tolerance of 0.01, such that the spline was within 0.01 of the simulated proportion resistant at each time-point. The spline fit was verified visually by plotting the spline and the model output (proportion resistant over time) for a subset of the 1000 simulations. Nineteen of the 1000 simulations could not be fit with a spline and therefore equilibriums and inflection points could not be found for those simulations. An equilibrium point was defined as the first time-point where the absolute value of the spline’s first derivative was < 1 × 10^− 9^ and remained < 1 × 10^− 9^ ten time-steps (1 h total) later. This equilibrium cut-off value had to be sufficiently small to correctly identify equilibriums in simulations that had relatively small changes in proportion resistant over time (hence consistently small derivatives), but still demonstrated clear behavioral patterns. For example, one simulation with increasing behavior may have a 20 percentage point change in proportion resistant from Day 2 to Day 90 while another simulation may have only a 5 percentage point change from Day 2 to Day 90. The equilibrium cut-off value was chosen by examining a subset of simulations visually and assessing the derivative value at the approximate visual equilibrium. The inflection point in a simulation was found by searching for the first pair of consecutive time points where the spline’s second derivative changed from positive to negative or vice versa. The first time-point of the pair was taken as the time of the inflection point. The starting proportion resistant (Day 2) was subtracted from the maximum levels and equilibrium levels in order to investigate relative maximum levels and relative equilibrium levels.

### Linear regression model building and selection

Three of the parameters (*N*_*start*_, *p*_*s*_, *start*_*s*_) were collinear with other parameters (Pearson correlation coefficient > 0.9) and therefore excluded from the regression models. *N*_*start*_ was a function of *N*_*max*_ so *N*_*start*_ was excluded and *N*_*max*_ was included in the regression models. The incoming proportion of antimicrobial-susceptible *E. coli* (*p*_*s*_) was a function of the incoming proportions of intermediate and resistant bacteria, since all three proportions must sum to 1. Similarly, the starting proportion susceptible (*start*_*s*_) was a function of the starting proportions of intermediate and resistant. Therefore *p*_*s*_ and *start*_*s*_ were also excluded from the regression models. Within each behavior mode, the input parameter values (*x*_*ik*_) were standardized for each of the remaining 26 parameters, *k*, to make them dimensionless and facilitate regression coefficient comparisons [20, 29], as follows:

$$ {\tilde{x}}_{ik}=\frac{x_{ik}-{\overline{x}}_k}{\sigma_{x_k}}\kern0.6em {\displaystyle \begin{array}{l}{\tilde{x}}_{ik}=\mathrm{standard}\mathrm{ized}\ \mathrm{value}\ \mathrm{of}\ \mathrm{parameter}\ k\ \mathrm{for}\ \mathrm{simulation}\ i\\ {}{x}_{ik}=\mathrm{non}\hbox{-} \mathrm{standardized}\ \mathrm{value}\ \mathrm{of}\ \mathrm{parameter}\ k\ \mathrm{for}\ \mathrm{simulation}\ i\\ {}{\overline{x}}_k=\mathrm{mean}\ \mathrm{of}\ \mathrm{parameter}\ k\ \mathrm{across}\ \mathrm{all}\ \mathrm{simulations}\\ {}{\sigma}_{x_k}=\mathrm{standard}\ \mathrm{deviation}\ \mathrm{of}\ \mathrm{parameter}\ k\ \mathrm{across}\ \mathrm{all}\ \mathrm{simulations}\end{array}} $$

Linear regression models were built using R 3.4.3 software [30] with the RStudio 1.0.136 (RStudio, Inc., Boston, MA, U.S.) user interface. Each pattern measure for each individual behavior mode was modeled separately as the dependent variable, resulting in 14 regression models. All input parameters (except for the three excluded for collinearity) were eligible to be independent variables. Thus the full regression models contained 26 input parameters as independent variables. Reduced models were built using two model selection techniques to obtain a best-fit, most parsimonious model. First, the models were fit with the ‘forward,’ ‘backward,’ and ‘both’ (stepwise) variable selection procedures (*step*, package *stats*) using the AIC (Akaike Information Criteria) as the fit criteria during the selection process. The reduced models suggested by the three variable selection routines were compared. Second, the best model subsets of up to 10 variables were identified using *regsubsets* (package *leaps*), which compares models of the same size with several selection criteria [31]. The models suggested by the variable selection routines and the best subsets were compared using BIC (Schwarz’s Bayesian Information Criteria), AIC, and adjusted *R*^*2*^. Of the models with similarly small BIC and AIC, and large adjusted *R*^*2*^, the most parsimonious model was selected as the best-fit model and was tested for model assumption validity.

Linear regression models must meet six assumptions in order to make valid predictions:

1. Observations are independent
2. Residuals follow a Normal distribution with a mean of zero
3. Linear relationship between dependent and independent variables
4. Homoscedasticity of residuals
5. Minimal or no multicollinearity of independent variables
6. Outlier observations do not drive the parameter estimates and predictions

We assessed whether each of the 14 best-fit regression models met these assumptions by examining the QQ plots, partial regression plots, standardized residuals versus fitted dependent-variable values, standardized residual histograms, Cook’s D plots, and calculating variance inflation factors. Violations of assumptions were addressed by transforming independent and dependent variables and excluding outlier output values when possible. Transformations included using polynomial terms for both independent and dependent variables in order to meet the assumption (3) of a linear relationship between dependent and independent variables. Input parameters were not re-standardized after transformations. In cases of large assumption violations, alternative regression models (e.g. Cox proportional hazard models) were considered for the behavior pattern measures.

We then further simplified each of the 14 best-fit models when possible by eliminating independent variables with small coefficients (< 0.005 for “level” models and < 240 for “time” models) as long as the model fit was not significantly altered (< 10% increase in BIC, < 2 percentage point decrease in adjusted *R*^*2*^, and no substantial change in residual plots) and coefficients of other parameters were generally unchanged (< 20% change). If removing a parameter did alter the model fit or other parameter coefficients, it was returned to the model. This process resulted in a best-fit, most-parsimonious model, which was again confirmed to meet linear regression assumptions.

### Cox proportional hazard model building and selection

Survival analysis was considered as an alternative model to linear regression for the ‘time to’ pattern measures (e.g. time to maximum proportion resistant). Survival analysis handles censored data, which can occur if a behavior pattern measure (i.e. ‘event’) does not occur before the end of the simulated time period. Behavior pattern measures can be considered right-censored if the ‘event’ could occur if the simulated process time or overall simulated time was extended. For example, some peaked-behavior simulations did not reach an absolute maximum proportion resistant during chlortetracycline administration (i.e. the proportion was still increasing when chlortetracycline administration stopped). Although these simulations did reach a local maximum at the end of chlortetracycline administration, if chlortetracycline administration was simulated for a longer time, those simulations may have reached an absolute maximum. Hence this pattern measure can be considered right-censored; specifically, it has end-of-study censoring. Cox proportional hazard models [32] were built (*coxph*, package *survival*) for the ‘time to’ pattern measures with the occurrence of a behavior pattern measure (e.g. maximum, equilibrium) as the ‘event’ and the time of the occurrence as the ‘event time.’ Both right-censored (absolute maximum is considered the event, which was not reached in some of the simulations that are then considered censored) and non-censored (local maximum is considered the event, which is reached in all the simulations and in some of those it is reached at the last time of chlortetracycline administration) Cox models were built. The standardized input parameters of the mathematical model were independent predictors. Stepwise selection based on the AIC was used to select the best-fit, most parsimonious right-censored and non-censored models. The best-fit model between the right-censored or non-censored was chosen based on the AIC and BIC, and for that model the assumption of proportional hazards was validated by testing for no interaction between Schoenfeld residuals and time (*cox.zph*, package *survival*) [33, 34]. Violations of the proportional hazard assumption were addressed by using a time-dependent coefficient for the violating parameter (i.e. specifying an interaction between time and the parameter that allows the coefficient to change continuously over time) or by stratifying the simulated time period into strata and making the parameter coefficient a step-function of the time strata (i.e. the coefficient is a constant value within each time stratum) [34, 35].

## Abbreviations

AIC: Akaike Information Criteria
BIC: Schwarz's Bayesian Information Criteria
CTC: Chlortetracycline
MIC: Minimum inhibitory concentration
